# Frailty nurse and GP-led models of care in care homes: the role of contextual factors impacting Enhanced health in care homes framework implementation

**DOI:** 10.1186/s12877-023-03742-3

**Published:** 2023-02-03

**Authors:** Zeibeda Sattar, Lesley Young-Murphy, Lynn Craig, Alison Steven, Gemma Wilson-Menzfeld

**Affiliations:** 1 Department of Health and Life Sciences, Northumbria University, Newcastle upon Tyne, UK; 2 Chief Operating Officer, North Tyneside Clinical Commissioning Group, Newcastle upon Tyne, UK; 3 Head of Quality, Safety and Development, North Tyneside Clinical Commissioning Group, Newcastle upon Tyne, UK; 4 Department of Nursing, Midwifery & Health, Northumbria University, Newcastle upon Tyne, UK; 5 Department, Nursing, Midwifery & Health, Northumbria University, Newcastle upon Tyne, UK

**Keywords:** Enhanced health, Care homes, Frailty nurse, Model GP-led, Model, Implementation, Recruitment

## Abstract

**Background:**

The Enhanced Health for Care homes (EHCH) framework is an innovative response to provide more proactive, preventative approaches to care for residents living in care homes. It involves co-producing a shared vision with primary care. As part of EHCH a UK clinical commissioning group supported GP’s in two localities to implement their preferred delivery approach involving a new Frailty Nurse-led (FN-led) model in care homes alongside an existing General Practitioner-led (GP-led) model. This paper focuses on implementation of the new FN-led model.

**Methods:**

A qualitative study design was adopted. Forty-eight qualitative semi-structured interviews were undertaken across six care home sites in a Northern locality: three implementing the FN-led and three engaged in an existing GP-led model. Participants included residents, family members, care home managers, care staff, and health professionals working within the EHCH framework.

**Results:**

Two overarching themes were generated from data analysis: Unanticipated implementation issues and Unintended consequences. Unsuccessful attempts to recruit Frailty Nurses (FN) with enhanced clinical skills working at the desired level (UK NHS Band 7) led to an unanticipated evolution in the implementation process of the FN-led model towards ‘training posts’. This prompted misaligned role expectations subsequently provoking unexpected temporary outcomes regarding role-based trust. The existing, well understood nature of the GP-led model may have further exacerbated these unintended consequences.

**Conclusion:**

Within the broader remit of embedding EHCH frameworks, the implementation of new FN roles needed to evolve due to unforeseen recruitment issues. Wider contextual factors are not in the control of those developing new initiatives and cannot always be foreseen, highlighting how wider factors can force evolution of planned implementation processes with unintended consequences. However, the unintended consequences in this study highlight the need for careful consideration of information dissemination (content and timing) to key stakeholders, and the influence of existing ways of working.

## Background

Finding ways to meet the health and wellbeing needs of older people (aged over 65) of increasing dependency is a major challenge worldwide [1–4]. With ageing populations globally [2, 5], policy makers, commissioners of services and care providers are constantly exploring a range of models in an attempt to enhance care [1, 6]. Residential facilities such as care homes or nursing homes are one approach to providing care for older adults who are no longer able to live independently, although the organisation and resourcing of care homes may differ across countries [7–9]. In 2021 figures showed 17,598 care homes (residential and nursing) operating in the UK, with around 490,000 residents [10]. The complexity of health problems occurring in the ageing population globally [9, 11] and in care home residents in England and Wales has notably increased over the past twenty years [4], with around 75% of residents admitted to care-homes in 2017 diagnosed with some level of cognitive impairment, multiple morbidity, frailty, sensory impairment, and functional decline [12]. However, a key priority remains the reduction of quality concerns and enhancement of care provision in care homes [1, 7, 13, 14].

Care home residents rely mainly on General Practitioners (GPs), community nurses and therapists for access to health care or referral to specialist services with such access mediated by care home staff [15, 16], however residents often report poor access to health services [17]. While GPs are amongst the most frequent health professionals to visit care homes and provide a key first point of contact for the majority of residents health needs, they have highlighted the complexity and difficulties they face in working to support older people in care homes [16]. A key aspect of these difficulties relate to the multiple relationships and wide network of people that GPs have to communicate with, compounded by high turnover rates of care home staff which impedes relationship formation and continuity of care [16].

In response to this situation, the ‘Enhanced Health in Care Homes’ (EHCH) framework was implemented in 2016, across the UK, as part of the ‘Vanguards Programme’ emanating from the NHS ‘Five year forward view’ [18]. The overarching ambition was to create a more integrated and sustainable health and social care system through new ways of working [18]. Six EHCH vanguard sites were commissioned across England [17]. The EHCH framework was proposed as a strategic approach, aimed at providing care home residents co-ordinated and proactive care, centred on the needs of individual residents, their families, and care home staff [17]. With seven core elements all requiring progress, the framework purported to champion a whole-systems approach by co-producing a shared vision and strong leadership [18, 19].

In 2017, Baylis and Perks [17] explored learning from the pilot EHCH vanguards via interviews with 30 individuals working across 15 local authority areas, reporting that the pilots prompted development of multi-disciplinary teams, the training of care home staff by a range of primary health care professionals, and promotion of integrated care. However, the need for a cultural shift to develop an understanding of changing roles and shared ownership of responsibilities for the care of residents was identified. Leadership, sensitivity to local contexts, care processes and relationships, delegation and development of trust, and investing time in clarifying aims and objectives through an inclusive process were highlighted as key areas [17]. While Baylis and Perks (2017 p.56) recommended that *‘all areas of England should develop enhanced health in care homes because doing so can bring significant benefits’*, they also noted that momentum would need to be maintained after the end of the vanguards in 2018 [17].

While new and changing roles emerged as part of the EHCH vanguards, the introduction of a new role in primary care is complex and intentions, involvement, communication, and acceptance are key to the implementation process [20, 21]. Furthermore in relation to the vanguards, Coleman et al., (2020) found inherent tensions between the bottom-up nature of the vanguard programme which encouraged development of enhanced care home initiatives relevant to local contexts (such as new roles), and the overall push for ‘generalisable’ frameworks suitable for wider roll out [18]. They suggested that the environment in which new initiatives are to be implemented should be considered and shaped with realistic goals, with a need for desired outcomes to be clear at the outset [18]. Indeed, the effectiveness, barriers, and facilitators for interventions integrating health and social care, such as EHCH, rely heavily on context [22], which it could be argued are complex and dynamic and ever-changing, and requires a receptive environment [23]. This resonates with much implementation science literature which highlights context as a key issue in the introduction and embedding of new initiatives in complex settings [24–26]. While some research into the role of GPs in care homes [15, 16] has been undertaken, there has been limited focus on the implementation of new ways of working and new care models in care homes [18, 22, 23, 27–29]. Against this backdrop the current study aimed to explore the embedding in care homes of a new Frailty Nurse-led [FN-led] model of care, alongside an existing General Practitioner-led [GP-led] model. This was prior to the subsequent implementation of Primary Care networks.

## Methods

### Design

A qualitative design was adopted drawing on principles of interpretivism. This approach enables the researcher to look beyond the descriptive to unpick and explore the process which emerge. This 18 month study is part of a larger project utilising a convergent parallel mixed-methods design [30]. This paper reports on the qualitative elements of the study only. This study was approved by the NHS IRAS ethical approval committee (Reference: 262720).

### Participants

This study is based in one Clinical Commissioning Group (CCG) area in Northern England. Four localities within the area were given additional funding to implement the EHCH project. Two models were proposed across the four localities to underpin the EHCH: FN-led and an existing GP-led model.

Both models were integrated as part of the local primary teams; the original FN model evolved into a training post (TFN -see findings) with role holders reporting to the Frailty team on a weekly basis as part of their training which also included studying appropriate modules at a local University (e.g. prescribing). Therefore, until they gained relevant qualifications and/or confidence they contacted their assigned GP practice for prescribing but carried out baseline observations as part of their diagnostics to inform GPs as required.

In this study, the term ‘care home’ is used to describe both nursing and residential homes. The sample of care homes for this study (*N* = 6) from a total of 30 care homes participating. The six care homes were purposively selected to generate variation in terms of model, size, and location of the care home (Table 1). All but one participating care home was dual registered (i.e., they were able accommodate both nursing and residential residents) and one operated as a nursing home only. In summary, three care homes from two localities volunteered to participate in implementation of FN-led model and a further three care homes from two localities remained with their existing GP-led model.

**Table 1:** Participant details

Job / Role	Number of participants
System leader	4
Primary Care Team	2
Care home manager	4
Care home resident	7
Family member	6
Frailty Nurse	3
GP	3
Senior carer / carer	13

Participants were eligible to take part in this study if they were: a resident or family member of a resident, care home manager, care staff, health professional working as part of this structure (including system leaders such as Directors, FNs, GP specialists, GP project support staff). Staff working in single, or multiple care homes were eligible for participation. All participants were over 18, had capacity to give full informed consent, and spoke/read English. Care home managers/staff identified participants with capacity for the research team. A total of 47 individuals participated (Table 1).

### Data collection

Research information was mailed to Registered Care Home managers, followed by a telephone call approximately 7 days later, allowing researchers to explain details and answer questions.

Where Registered Care Home managers agreed for the home to participate, information was then distributed to relevant staff, residents, family members, and health professionals. Care home participation was separate to individual participation. All individuals were able to decline participation without any impact on their work/care. Those indicating interest were sent consent forms and interviews arranged. To develop a more detailed understanding of role development, FNs and FN Leads were interviewed at three separate time points, beginning, middle and end across the project. This time period allowed for FNs to build relationships and trust.

Drawing on existing literature, multi-disciplinary team expertise, and prior experience, a semi- structured interview topic guide was developed for all stakeholders, consisting of several broad open-ended questions covering; ongoing activity regarding the EHCH framework implemented in their care homes; how this new way of working was organised; discussion of any changes to care (proactive and reactive), exploration of benefits and barriers to the EHCH framework. Participants were encouraged to talk freely and raise issues they felt were of importance. Most interviews were face-to-face (*n* = 39) with a small number via telephone (*n* = 8). All interviews were audio recorded and transcribed verbatim.

Reflexive, inductive thematic analysis was undertaken following the steps outlined by Braun and Clarke [31–33]. The analysis was informed by the seven key themes identified within the NHS framework [19]. ZS analysed all transcripts. Initially, the analyst immersed themselves within the transcripts before generating initial codes and subsequent themes [31]. Analysis aimed to go beyond simple description of participants experiences, to abstract and unpick the bigger picture regarding what happened during the implementation process. To enhance data analysis rigour, sample transcripts (*n* = 6) were circulated to team members (ZS, AS, GWM) who undertook initial individual coding of transcripts taking into consideration the project aims, commonalities, discrepancies, unusual and unexpected issues. This was followed by two meetings to discuss and agree codes which were reapplied to all transcripts by one team member (ZS). A final team meeting discussed, interrogated, and agreed the final themes. All methods were carried out in accordance with relevant guidelines and regulations.

## Results

Two themes were generated from the data analysis: Unanticipated implementation issues and Unintended consequences, each with two sub-themes (Table 2).

**Table 2:** Summary of Results

**Unanticipated implementation issues**	Recruitment challenges Additional support and education
**Unintended outcomes**	Misaligned role expectations Trust and relationship building

### Unanticipated implementation issues

#### Recruitment challenges

A key part of the EHCH implementation process was recruitment of staff to the newly developed FN role. However, stakeholders found themselves facing unanticipated challenges in recruiting staff at the desired level (UK NHS Band 7 level) and failed to fill these advertised roles.

*‘We advertised for [x number of] nurses … but it very quickly became apparent that the workforce wasn’t there, at that band seven, community matron-type level. … then over a shortish period of time we went out for further recruitment’* (FN-led Model, Participant 1: System Leader)

Consideration of these recruitment issues, alongside local budgets, and programme timescales, resulted in a strategic decision to re-advertise as ‘Trainee’ Frailty Nurse (TFN) posts at a lower level (UK NHS Band 6). Thus, the posts evolved to be training positions in which appointees could develop and grow to fit the local context. These unanticipated contextual factors changed the model from a FN-led model to a TFN-led model and prompted a series of unintended programme consequences.

Senior care staff and care home managers recalled receiving information about the implementation of EHCH and the planned appointment of FNs. They were enthusiastic, viewing the FN-led model as a positive change.

*‘We had great expectations, because we were finding a lot of problems with contacting doctors’* (FN-led Model, Participant 15: Care Home Manager)

However, care staff seemed unaware of the subsequent change from FNs to TFNs and the additional educational element to the role that was needed because of these changing posts. This ‘mismatch’ of information disseminated originally and the evolution of the role to a training position led to unintended consequences, uncertainty, and confusion regarding the role parameters of TFNs. There was also a perception that recruitment of staff to TFN roles could have involved greater targeting of existing staff from within the care homes themselves.

*‘Whoever thought of the scheme, should maybe have gone round all the trial homes that they’re trying out in, and saying have you got anybody that you think fits the bill, that might like to do this role?’* (FN-led Model, Participant 15: Care Home Manager)

#### Additional support and education

Due to the evolution from the FN-led to TFN-led model, various support mechanisms were implemented to assist the new trainees in gaining relevant skills and knowledge and in developing the role. Extra support was instigated both in practice settings and through access to university courses leading to the required qualifications.

GP frailty specialists and specialist frailty leads were recruited as part of the TFN support team and facilitated individual personal development plans, peer support mechanisms alongside delivering weekly education sessions to the TFNs. Whilst undertaking this training, TFNs were limited in the clinical activity, such as assessments, they could undertake.

‘O*nce I’ve done my clinical skills – my skill level will be higher than that of the current nursing staff. And I will then go and listen to the chests and say, yeah, I’ll get the GP to prescribe some antibiotics. But, at the minute, I don’t have any more skills than the nursing staff’* (FN-led Model, Participant 9: TFN)

TFNs were regularly mentored and for the first 6 months, were supported by the FN Lead to develop their role in context. This was also linked to preparing for future statutory national drives regarding specialty care requirements for the ageing cohort these nurses were working with. This support was helpful to all TFNs and the wider team, given they came to the posts without formal frailty training.

‘ … *Only one of them was from that [frailty] background. But they all have been looking after older people … It was getting one-to-ones, getting team meetings … Making them be safe from a nursing perspective. And … the way that they worked in this medically driven primary healthcare federation world’* (FN-led Model, Participant 11: Primary Care Team)

### Unintended consequences

#### Misaligned role expectations

The evolution of the ‘trainee’ role led to misalignment and confusion regarding what the FN role entailed, leading to temporary unintended consequences. There was a general uncertainty about the TFN role, which was often compared to a GP role. The narratives below illustrate staff perceptions regarding role expectations and TFN duties, and it is interesting to note that the care home manager in the second quote seems not to understand, or is unaware, that the TFN is already a registered nurse:

‘*The very first time I [met the FN I] didn’t really understand what [the role] was. The next time I think it was… She explained what it is... that it’s not a GP, but it’s sort of a high… Like, she used to be a nurse... But then they do something like 98% of what a GP does or something’* (FN-led Model, Participant 22: Care staff)

*‘[The FN] cannot put in any real input into the home…It’s a misleading perception to everybody…Frailty Nurses…The contents don’t do what the tin says. And for me… I find it misleading because if you went into a hospital and someone had on their badge that they were a nurse, and they were actually a carer... You know, they were working towards being a nurse, and you thought that person was a nurse - how would you feel about that?’* (FN-led Model, Participant 15: Care Home Manager)

Care staff from the TFN-led model felt misinformed about the role, remit, and responsibilities of the TFNs, which made them feel less trusting of decisions and judgments regarding residents’ care. Staff were mostly unaware of the educational and developmental requirements within the trainee’s role, and this led to unintended consequences of uncertainty and confusion. Some care staff expressed a preference for a GP-led model, as they perceived GPs as having the ability to provide immediate treatment and being unable to refuse requests to visit residents.

*‘I don’t think [the FN has] relieved us from doing anything. Now, I think if we had a GP coming into the home every day - oh, what a fantastic difference that would make to us. Because we could say to them, oh, we’ve got so-and-so, who we think is a bit poorly... Can you have a look at them? Now, I think that would be fantastic’* (Participant 16, Care Staff: FN-led Model)

The lack of understanding led to unintended consequences of mistrust regarding the (T)FN role, despite efforts of system leaders to provide a dedicated team to train the TFNs and disseminate information about their remit and development. Conversely, positive experiences of the existing GP-led model were perceived to be directly linked to GP credibility and familiarity with the GP role.

#### Trust and relationship building variable

Relationship building was pertinent for resident care, staff development, and multi-agency relationships. Many stakeholders felt that the overall EHCH framework (covering both FN-led and GP-led models) helped to facilitate relationship building through (optional) care home alignment, i.e., supporting care home residents in each care home to join a specific GP practice. Its advantages included consistency of the FNs, GPs, or Practice Nurses visiting care homes, although this alignment took commitment from all stakeholders to execute.

*‘I know my client group. I know when they’re well, and I know when they’re not well. And because I know them and their family, I think I’m better positioned than the GP so that I can feedback’* (FN-led Model, Participant 9, FN)

‘*I can see if somebody is deteriorating. You know, if somebody with dementia is getting suddenly more confused - a GP that doesn’t know them might think, oh, they’re just getting worse dementia. Whereas I would know that probably there’s a delirium there. And... And it may need to be actioned with some investigations or, you know, sort of, checking things out. It’s great continuity. It means we can, as we’ve been saying, be more proactive with care’* (GP-led Model, Participant 25, GP)

Whilst the EHCH framework supported consistency through GP alignment, some inconsistencies were still experienced, particularly when GPs visited care homes on an ad-hoc basis.

*‘Because we used to get various GPs, and they didn’t know who the individual [resident] was.…So, now we’ve got a regular GP, we’re all on the same wavelength’* (GP-led Model, Locality B, Participant 37: Care Staff)

Care staff, residents, and their families felt that relationships were developed because of regular and consistent visits made by the TFNs or GPs in each model.

Alignment to GP practices was encouraged but was not mandatory and it was sometimes difficult for care staff to engage with those ‘outlying’ GP practices where residents had not moved to the aligned Gp practice. Care for these residents was particularly difficult as aligned GP practices were unable to access patients records if that resident was not registered in their practice.

*‘Not all of the residents are part of the surgery who’s aligned to us. So, we’ve got three other surgeries who... We find very difficult to get them to come in’* (GP-led Model, Locality B, Participant 12: Care Staff)

Relationship building was also central to staff development through improved information sharing and proactive care; central components of the EHCH framework. In the narrative below a TFN explained how their alignment to one care home enabled them to make change and reduce falls. This was made possible through familiarity with the care home, its staff, and residents.

*‘You can see through the investigation of them if there’s any recurrent places that they fall. Or recurrent reasons. And the lounge was one of… It tended to be one of the main areas… And made the biggest impact…stuff’* (Participant 9, FN-led model)

Not all information sharing was positive, sometimes a lack of consistency in communicating information which was felt to impact resident care negatively.

‘*If [the FN] doesn’t document, then that could lead to problems. So, I think she needs to keep up with documentation when she’s done anything at all, to writing the doctor’s notes, the MDT notes… the family notes. Or if there’s something she needs to handover – put it in the book for the nurse. Or leave a note for the nurse. You know if it’s not written down, it’s not done’* (Participant 15, Care Staff: FN-led Model)

This documentation was critical for residents’ care. As part of the GP-led model, GPs also suggested that frequent care staff changes, including Registered Managers, negatively impacted establishing key information about residents, for example, in Emergency Health Care Plans (EHCPs). It was felt that this was due to a lack of time care staff had spent with residents and this was why some care staff could not provide a comprehensive account of resident issues.

In relation to the existing GP-led model, the care staff felt they had strong relationships with GPs, residents, and their families, and the regular visits empowered staff. For example, care staff were trained to adhere to the ‘*watch and wait*’ policy and take responsibility for closely monitoring residents and recognise illness early.

‘*[The watch and wait policy] quite a complex issue, the carers wouldn’t recognise early illness. So, then, the person would be quite poorly by the time they got a GP to come out and visit them’.* (Participant 10: FN, FN-led model)

Relationship building across the multi-disciplinary team also strengthened communication with residents and their families, as they fulfilled their requirements to complete EHCH documentation e.g., end of life care plans. There were reports of enhanced care for residents within both models. Care staff explained that a close working relationship with GPs helped them to understand their roles and responsibilities, but more significantly they were preparing observations and acting sooner to resident care needs because they were aware of GPs visiting regularly.

*‘So, working with them a bit closer, and more regular, it makes you understand what they’re do and what they can and can’t do’* (GP-led Model, Participant 37: Care Staff)

Unlike with the existing GP model, an issue that impacted relationship building and affected trust was the misalignment of the TFN role, as discussed above. Despite wider strategic efforts to promote preventative care and support TFNs within care homes, care staff were uncertain, and somewhat untrusting, of the trainee role. Judgements were questioned, and this created tensions between the care home and TFNs.

*‘Yes, because we know what’s going on... But she seems to be looking after the wrong ones, instead of concentrating on the ones that are really poorly, you know. I don’t know what more I can say, really, because…’* (Participant 21, Care Staff: FN-led Model)

Uncertainty and mistrust were exacerbated by a lack of awareness or understanding regarding the evolution from the autonomous FN role initially portrayed to that of trainee.

Despite the ongoing issues, the operationalisation and implementation of both models aimed to support proactive care through alignment that ultimately led to relationship building. As a GP describes below, the benefits outweighed the weaknesses in the framework because they were developing a close working relationship with the care staff through policy, education, and guidance.

*‘I think the staff really appreciate it. And there is an opportunity for a bit of education and support of them. Because they have a very heavy burden as well. You know, they’re dealing with some very poorly patients’* (GP-led Model, Participant 25: GP)

## Discussion

This qualitative study aimed to explore the embedding of EHCH framework via the development of an FN-led model, however unanticipated contextual and relational issues impacted on the process and outcomes. Implementation and quality improvement science approaches acknowledge the importance of context in complex settings such as care homes, offering useful lenses for considering findings [13, 26, 34–37]. Normalisation process theory (NPT) [35, 38] has been widely used to illuminate process and context issues in the implementation of new practices [35, 39, 40] including in care homes [41]. NPT consists of a framework of four constructs core to normalisation: Coherence-sensemaking; Cognitive Participation-working out participation; Collective Action – doing it; and Reflexive Monitoring- appraising the effects [35]. The Alberta Context Tool [42] which measures organisational context via 8 domains (including resources, communication patterns and interactions) has also been used to assist mapping of contextual elements influencing the implementation of care delivery initiatives [34].

A challenge to implementation of the new FN model was recruitment of staff at the desired level (UK NHS Band 7) because of a lack of sufficiently experienced or skilled applicants. Therefore, the initiative evolved with the role being revised to a ‘training’ position FN-TFN (UK NHS Band 6) necessitating development of a support package for the TFNs. Although in a different context, Nancarrow et al., (2015) identified several mechanisms to facilitate the implementation of a trainee role with positive outcomes. These mechanisms included supporting existing staff, clearly defined role and delegation boundaries, consultation and engagement and a targeted recruitment approach via a traineeship approach [30]. This ‘traineeship’ approach, supported by the Frailty Capability framework [43] may be a useful consideration for others.

The change from qualified FN to trainee also led to confusion for some key stakeholders as information had already been disseminated regarding appointment of fully qualified FNs, thus raising expectations. In addition, the FN-TFN role was implemented alongside an existing GP-led model, which may have exacerbated a ‘rippling effect’, regarding role expectations, relationship building and trust issues. Thus, for some the project perhaps no longer made sense or had ‘coherence’ and their cognitive participation may have waned [40]. Such confusion may have also prompted a sense of conflict between their understandings of the original goals of a FN post and those of the trainees who were appointed. Consistent understanding of goals (i.e. coherence around roles and responsibilities) may result in higher levels of work engagement (collective action) and increase work motivation and job satisfaction [44]. It is also crucial to recognise role assimilation to better align staff with goals and increase the commitment (and collective action) needed to enable role stability [45, 46].

Coleman et al. (2021) used Matland’s (1995) ambiguity-conflict model to explore large scale top-down policy implementation with a focus on Vanguard research, concluding that the model indicates the need for programme goals and potential conflicts to be raised and considered [18]. We suggest that Matland’s model could provide a useful tool in identifying and accounting for would-be conflicts when developing goals (e.g. between expectations for fully qualified vs trainee posts) [18]. In addition, drawing on the ACT tool and stakeholder consensus groups, Bunn et al. (2020) recently analysed research from Vanguard areas and developed a ten-question framework for promoting conversations between stakeholders around implementation of interventions in care homes [34] which may also be useful in future initiatives. A ‘launch’ strategy to factor in ‘timing and content’ of information and the feasibility of delaying information dissemination until recruitment is complete could be also considered when introducing new roles and may mitigate unintended consequences.

As seen in this study unintended implementation issues can impact on relationships and trust, as information ‘trickles down’ amongst care home management staff through formal and informal interactions [42]. Perceptions of insufficient communication after the decision to change the role definitions to that of trainee appeared to create tensions between professional boundaries [21]. Interpersonal trust is reciprocal and may be difficult to re-gain once broken and is therefore important to consider. Trust is informed through relationships, however as Bunn et al. (2020) noted, relational working requires support and time to develop [35]. Another structured process that may support such challenges and improve trust are quality improvement collaboratives (QICs); these bring together multidisciplinary teams in a structured way to improve care quality. For example, in a study by Devi et al., 2021 care staff stated that that people did not take notice of what they had to say because they were not employed by NHS staff. Thus recruiting collaborative members experienced in working in care homes to team meetings and discussions may support goal clarity (coherence), relational working and reduce conflict [13], thus potentially mitigating challenges such as those faced by TFNs. The use of implementation models may have also helped to establish key challenges and plan mitigation measures from the outset.

Although introduction of the FN can be viewed as an example of direct role substitution [13, 18, 47, 48] modifying the role to that of trainee altered intended skill mix dynamics. While skill mix can enhance quality of patient care it takes effort to implement and maintain [21, 47, 48]. This highlights the difficulties for strategic leaders and commissioners who may wish to appoint to a new role but cannot completely know the pool of staff from which they are trying to recruit, or all the nuances of the wider workforce context. The shortage of supply of experienced, qualified nurses and high turnover of nursing staff in adult services are longstanding issues [49], however this should not preclude the development of new roles and models of care such as the EHCH framework. Indeed, despite the initial setbacks a skill mix can enhance the quality of patient care [19, 21, 30]. New roles such as the FN may be attractive to staff looking for new challenges or career change. Whilst previous vanguard studies also report specialist primary care role developments have been challenging [19], we were unable to find studies that report on recruitment difficulties, making this study distinctive.

Despite the implementation issues this study has indicated, in line with the EHCH [19] aims, that the regular weekly visits from the TFNs and GPs in the care homes (including staff, residents, and their families) allowed for consistent and continuous care of residents, particularly proactive care, continuity of care, and advance care planning, due to a better understanding of the resident health needs. Two further core elements were identified as being effective in the EHCH framework [19] from national vanguards: “*Joined-up commissioning of health and social care, and collaboration across the health and social care system (as well as between individual care homes, GP practices and community teams)*” and “*workforce development, including consideration of training needs and new roles working across organisational boundaries*” because of care home alignment. This is a common theme across multiple vanguard evaluations which supports the importance of multidisciplinary, partnership working and good relationships between care home staff and other professional groups [16, 50]. Cook et al., (2017) reported the importance of relationship building between care home residents and staff, as staff used multiple forms of information to inform decisions about the management of residents’ care [43]. This baseline understanding of the person as a whole, and intuition of changes, enabled individuals to provide proactive care [43]. In addition, providing nurturing opportunities to new roles, for educational purposes, building social capital may result in better outcomes for key stakeholders involved [51] highlighting that the project team effectively responded to the changes.

## Conclusion

The EHCH framework ensures care home residents receive co-ordinated, proactive care, centred on the needs of individual residents, their families, and care home staff. This framework was implemented and evaluated in this current study with a view to reflect on future commissioning intentions and national developments with the advent of Primary Care Networks (date). The findings highlighted the complexity of the EHCH framework focussing on the new TFN role model, and its implementation strengths and weaknesses. Unanticipated implementation issues, namely recruitment challenges and additional support and education were identified. In addition, unintended consequences were identified as a result, misaligned expectations and trust and relationship building variable. Despite these challenges, data from this novel 18 month study illustrated that over time, as relationships seemed to develop between TFNs and care staff, the role and associated remit became more accepted and understood.

## Data Availability

Data can be made available if someone request the data from this study. Please email zeb.sattar@northumbria.ac.uk
